# Symptomatic Plantar Osteochondroma of the Third Metatarsal With Toe Flexion Deformity: A Case Report

**DOI:** 10.7759/cureus.100781

**Published:** 2026-01-04

**Authors:** Salah Al Kholaki, Fadi Nader, Georges F Bassil, Zied Missaoui

**Affiliations:** 1 Orthopedic Surgery, Grand Hôpital de l'Est Francilien, Meaux, FRA; 2 Orthopedic Surgery, Grand Hopital de l'Est Francilien, Meaux, FRA; 3 Orthopedic Surgery, Lebanese University Faculty of Medicine, Beirut, LBN

**Keywords:** benign, metatarsal bone, osteochondroma, plantar face of the foot, tumor

## Abstract

Although osteochondroma is the most common benign bone tumor, it rarely affects the foot, particularly the plantar aspect of the metatarsals. Plantar lesions are clinically significant due to their involvement in weight-bearing and potential to cause pain and deformity. We report the case of an 18-year-old male presenting with a painful plantar mass of the left foot arising from the distal third metatarsal. The lesion had been present for three years with rapid growth over the preceding four months, resulting in plantar pain, toe flexion deformity, and functional limitation. Clinical examination revealed a well-circumscribed, hard mass measuring approximately 2 cm. Imaging studies demonstrated a calcified exostosis originating from the plantar surface of the third metatarsal. The lesion was surgically excised through a plantar approach. Histopathological examination confirmed the diagnosis of osteochondroma. At the one-year follow-up, the patient was pain-free, with no evidence of recurrence and restoration of normal toe motion. This case highlights a rare plantar localization of a third metatarsal osteochondroma and emphasizes the importance of recognizing atypical sites to ensure timely surgical management and optimal functional outcomes.

## Introduction

Osteochondroma is a developmental lesion rather than a true neoplasm, representing 20%-50% of benign bone tumors and 10%-15% of all bone tumors [1,2]. It occurs most commonly in the metaphyseal region of long bones, such as the distal femur, proximal tibia, and proximal humerus, as a result of abnormal endochondral ossification. Solitary osteochondromas typically appear in childhood and adolescence and can manifest as sessile or pedunculated outgrowths covered by cartilage, visible on radiographs. Involvement of the foot is uncommon, accounting for fewer than 1% of reported cases [1]. Within the foot, osteochondromas most often affect the dorsal aspect of the metatarsals, while plantar localization is particularly rare. Lesions arising on the plantar surface are clinically significant due to their direct involvement in weight-bearing, which may lead to pain, altered gait, toe deformity, and functional limitation.

Osteochondromas originating from the third metatarsal are exceptionally rare, with only a limited number of cases reported in the literature. When located plantarly, these lesions may compress flexor tendons and adjacent structures, resulting in progressive deformity and impairment of daily activities. We report a rare case of a plantar osteochondroma of the third metatarsal in a young adult, highlighting its functional impact, diagnostic challenges, and successful surgical management.

## Case presentation

**Figure 1:**
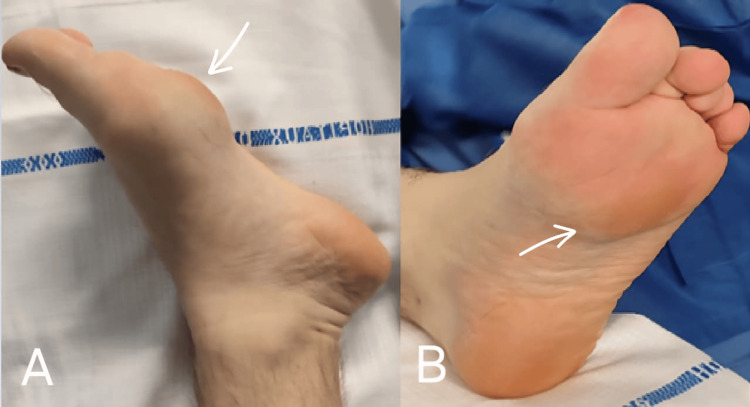
Clinical image of the osteochondroma on the plantar aspect of the foot (A) and its effect on the flexion of the second and third toe (B).

An 18-year-old male with a body mass index of 22.9 kg/m² presented with a painful mass on the plantar aspect of his left foot. The mass was first observed three years earlier but had shown a rapid increase in size during the past four months. This progression led to pain, deformity of the plantar surface, and flexion of the third and fourth toes due to tendon retraction (Figure 1), making daily activities increasingly difficult.

Clinical examination

On examination, a 2 cm firm, well-circumscribed mass was noted on the distal plantar aspect of the third metatarsal. It was non-compressible, fixed to the underlying bone, and non-mobile, with no adherence to the overlying skin. Its bony consistency and fixation made soft tissue lesions such as plantar fibromatosis or calcified soft tissue masses unlikely. The mass was painful upon palpation but showed no signs of infection or overlying skin changes.

Imaging findings

**Figure 2:**
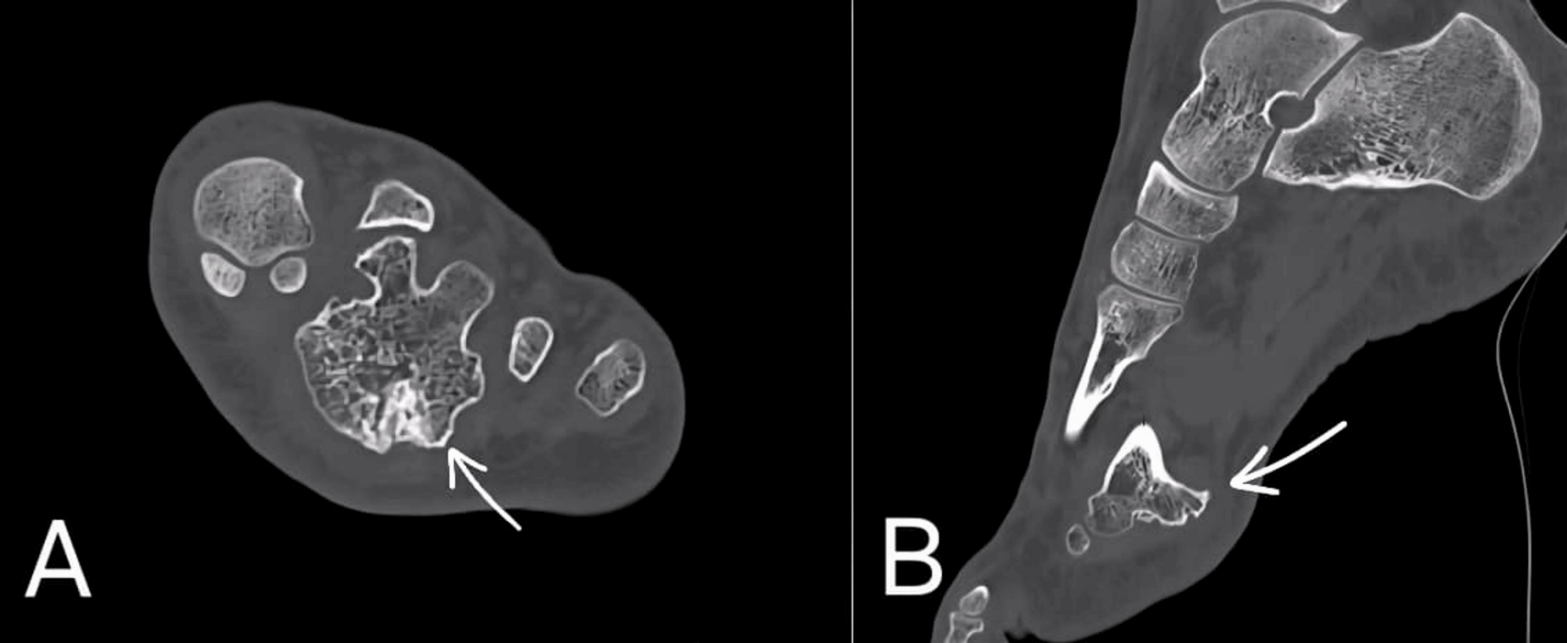
Coronal (A) and sagittal (B) CT images of the left foot showing a plantar exostosis arising from the distal third metatarsal (arrow), in close relation to the second metatarsal.

Radiographs revealed a well-defined, calcified mass located at the distal plantar aspect of the third metatarsal, extending toward the second metatarsal. CT scan demonstrated an exostosis originating from the third metatarsal bone, measuring 3.2 cm (Figure 2). The lesion was in contact with the second metatarsal bone, with posterior cortical thickening.

Surgical approach

**Figure 3:**
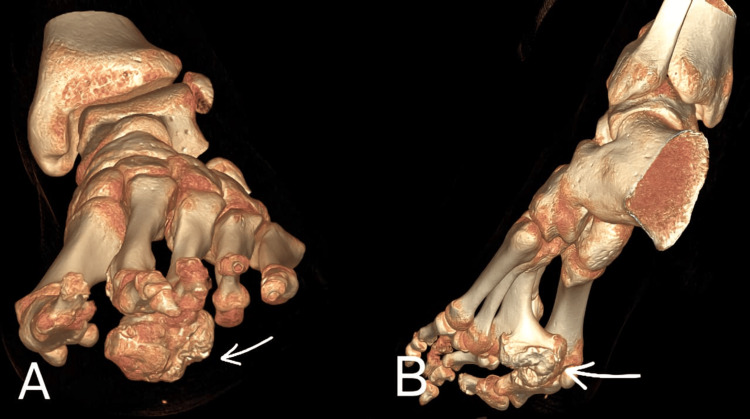
Three-dimensional CT reconstruction of the left foot illustrating the plantar origin and extent of the osteochondroma.

A longitudinal plantar incision of approximately 4 cm was made directly over the lesion (Figure 3) to allow direct access to the lesion’s plantar origin and complete excision while minimizing bone resection and avoiding extensive manipulation of adjacent metatarsals that would have been required with a dorsal or medial approach. Careful layer-by-layer dissection was performed to protect the flexor tendons and surrounding neurovascular structures, allowing direct exposure of the bony mass. It was encapsulated and required careful dissection from surrounding structures. The Tessier maxillofacial osteotome was used due to its thin profile and precise cutting edge to excise the lesion, and a drain was placed to prevent hematoma formation postoperatively. The mass was compressing the flexor tendons.

Histopathological findings

**Figure 4:**
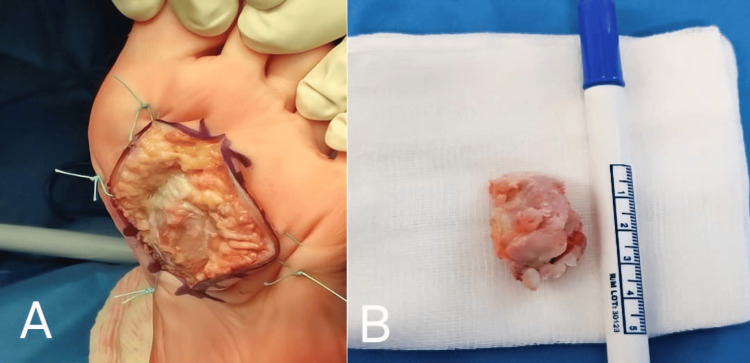
Intraoperative photograph demonstrating the direct plantar approach (A) and exposure of the osteochondroma after protection of surrounding soft tissues, and the excised mass (B).

The excised mass (Figure 4) measured 3.5 × 2 cm and consisted of a hyaline cartilage cap measuring less than 5 mm, showing no cellular atypia, mitotic activity, or features suggestive of malignant transformation, with mature trabecular bone centrally. Histological evaluation confirmed the diagnosis of osteochondroma. The difference between the clinically estimated size (2 cm) and the excised specimen dimensions (3.5 × 2 cm) reflects partial deep plantar extension of the lesion, not fully appreciable on physical examination.

Follow-up

**Figure 5:**
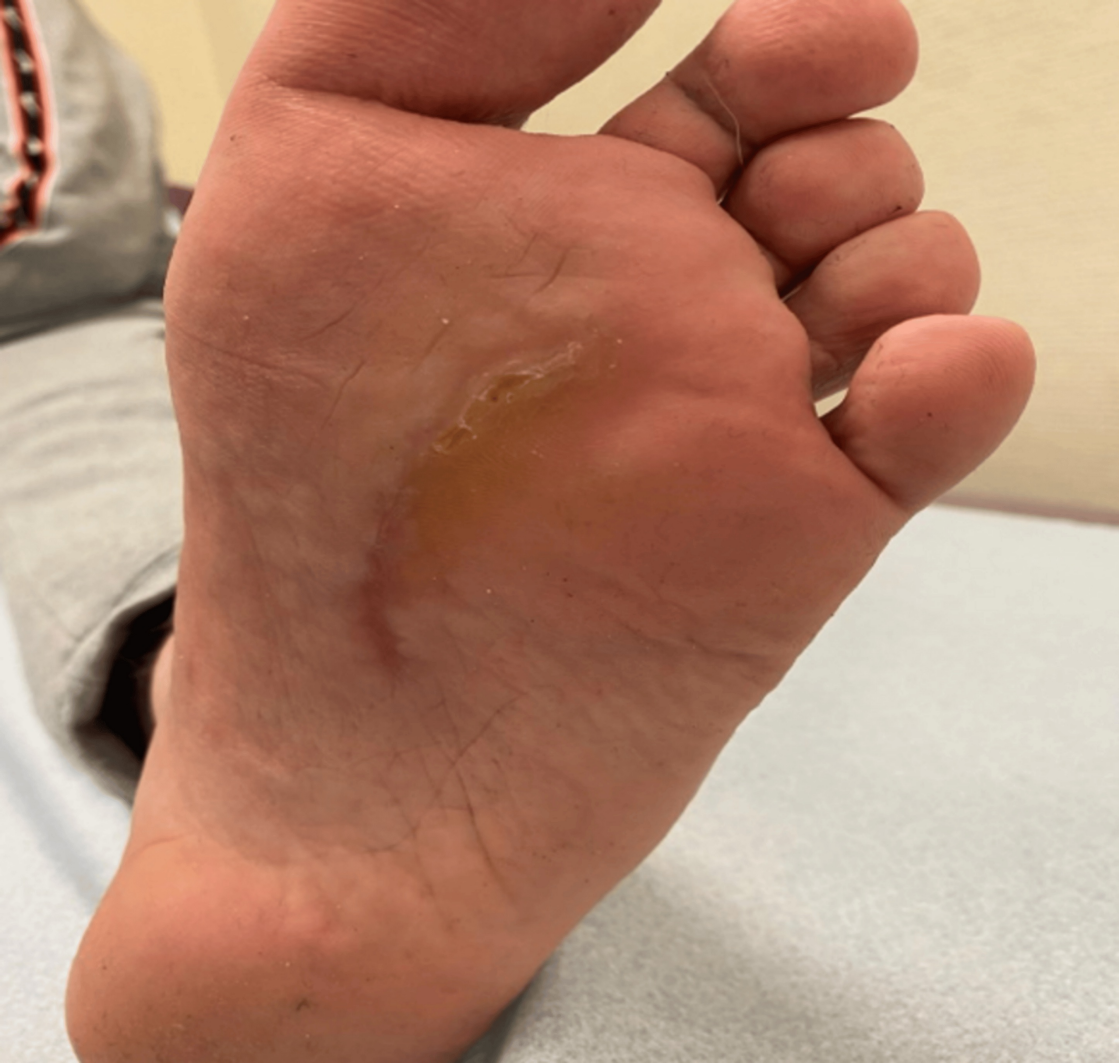
Healed incision after one month postoperatively.

**Figure 6:**
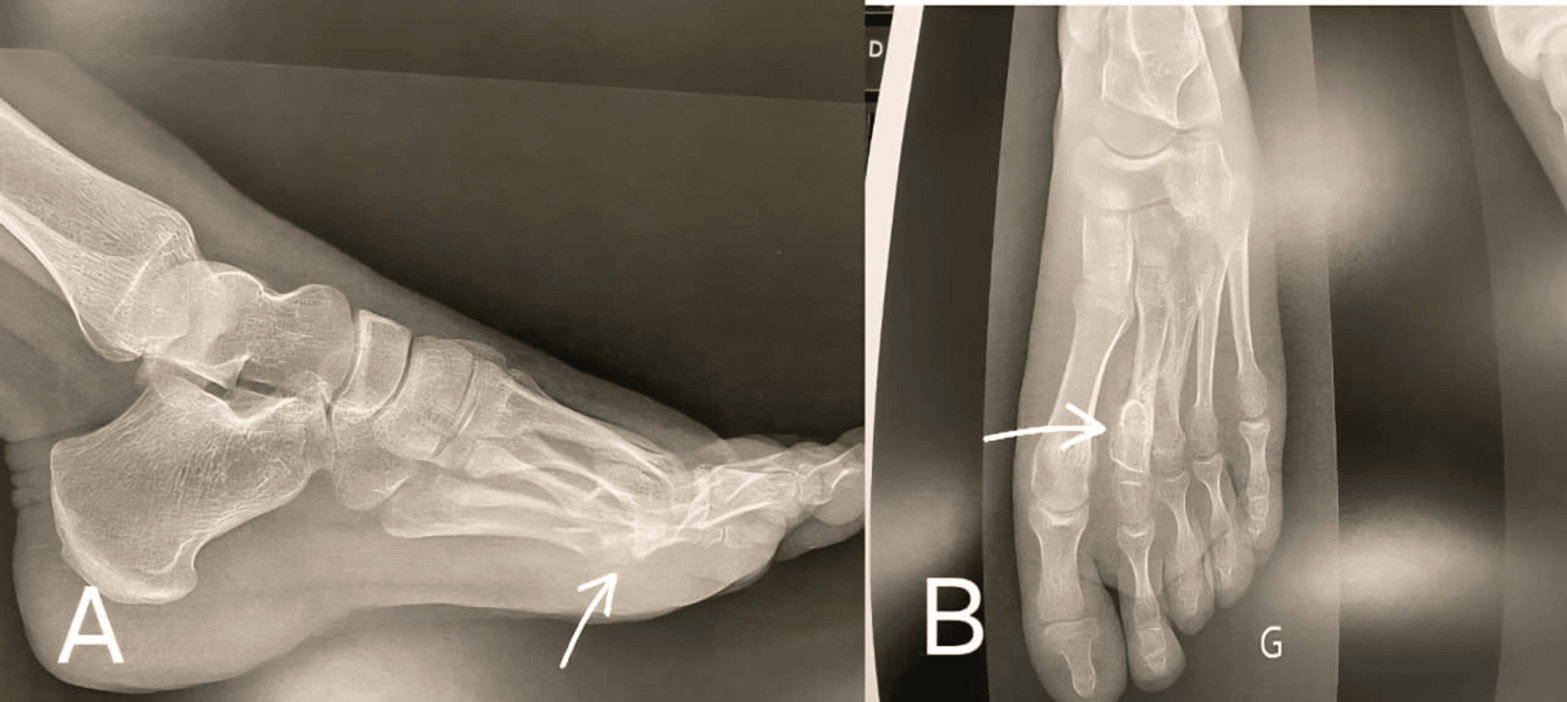
Lateral (A) and anteroposterior (B) radiographs of the left foot at one year postoperatively. White arrows indicates the location of the old osteochondroma.

At one month postoperatively, the patient reported mild residual discomfort (Visual Analog Scale (VAS) pain score: 2/10). The surgical incision was fully healed (Figure 5), and partial correction of the toe flexion deformity was observed, with improved passive extension of the third and fourth toes. At three months, the patient was pain-free (VAS score: 0/10) and had returned to normal daily activities without limitation. At 12 months, clinical examination showed complete resolution of pain, normal toe alignment, and full range of motion. Radiological follow-up using plain radiographs demonstrated no evidence of recurrence (Figure 6).

## Discussion

Osteochondroma constitutes the most common benign bone tumor, accounting for 36%-41% of all cases. Although rare in the foot (fewer than 1% of cases), the symptoms range from being asymptomatic to causing pain, deformity, or neurovascular compromise. Imaging modalities such as MRI are crucial for assessing cartilage cap thickness and ruling out malignant transformation, particularly if the cap exceeds 15 mm [2-4].

The present case is notable for the rare plantar localization of an osteochondroma arising from the third metatarsal. Unlike the more commonly reported dorsal metatarsal lesions, plantar tumors directly affect weight-bearing and may lead to pain, altered gait, and secondary toe deformities due to flexor tendon compression, as observed in our patient.

Although MRI is commonly recommended to assess cartilage cap thickness and exclude malignant transformation, it was not deemed necessary in this case because CT imaging clearly demonstrated a benign exostotic lesion with cortical and medullary continuity, a thin cartilage cap on histopathology, and no clinical or radiological features suggestive of malignancy.

Complete surgical excision remains the treatment of choice for symptomatic osteochondromas. In this case, a plantar approach allowed direct access to the lesion’s origin, facilitating complete resection while avoiding unnecessary osteotomy or manipulation of adjacent metatarsals. Similar favorable outcomes without recurrence have been reported in previous plantar and metatarsal osteochondroma cases when complete excision was achieved. When performed thoroughly, it is associated with excellent outcomes and only rare recurrences [5-10].

Comparison of different case reports in the literature with our study is presented in Table 1. Compared with previous cases, the present one is unique due to its plantar location on the third metatarsal and its associated functional toe deformity.

**Table 1:** Case reports reported in the literature compared to our study. NA: not available

Study	Year	Age (years)	Site	Treatment	Recurrence
Estil et al. [5]	2013	49	Medial plantar arch	Excision	No
Patil et al. [6]	2016	13	Fourth metatarsal dorsum	Excision	No
Rodríguez Rodríguez et al. [7]	2018	10	Second metatarsal dorsum	Excision	No
Harna and Maini [8]	2020	65	Fifth metatarsal dorsum	Excision	No
Mujahed et al. [9]	2021	11	Second metatarsal plantar	Excision	No
Sajeev et al. [10]	2022	21	Second and third metatarsal dorsum	Excision	No
Our case	2024	18	Third metatarsal plantar	Excision	No

The strength of this report lies in the detailed clinical, radiological, surgical, and histopathological correlation of a rare presentation. Limitations include the single-case design and the absence of validated foot-specific functional scores. Nevertheless, this case highlights the importance of considering osteochondroma in the differential diagnosis of plantar forefoot masses and supports early surgical intervention to restore function and prevent progressive deformity.

## Conclusions

This case highlights the diagnostic and therapeutic challenges of a rare plantar osteochondroma arising from the third metatarsal. Due to its weight-bearing location, such lesions may cause pain, toe deformity, and functional limitation. Careful clinical and radiological evaluation is essential, and complete surgical excision through a tailored approach can result in excellent functional outcomes with a low risk of recurrence when performed thoroughly.
